# Supplementary material for: Sensitivity to social norm violation is related to political orientation
Source: PLoS One. 2020 Dec 1;15(12):e0242996. doi: 10.1371/journal.pone.0242996 (PMC7707570; doi:10.1371/journal.pone.0242996)
Supplement: S2 File. Questionnaire — (DOCX) [file pone.0242996.s002.docx]

**CALD political questionnaire**

| **TAXES (economic axis)**  Taxes must be lowered for everyone when the state has the means and raised for everyone when necessary. (3)  General tax cuts are needed to allow businesses and individuals to invest more money in the economy in order to create more jobs. (5)  It is necessary to lower the taxes which weigh on the less wealthy, and to increase them on the wealthiest people or companies to use solidarity and to give to the State the means to finance public services. (1) | ***LES IMPOTS (axe économique)***  *Il faut baisser les impôts pour tous quand l'Etat en a les moyens, et les augmenter pour tous quand c'est nécessaire. (3)*  *Il faut une baisse générale des impôts pour permettre aux entreprises et aux particuliers d'investir plus d'argent dans l'économie, afin de créer davantage d'emplois. (5)*  *Il faut baisser les impôts qui pèsent sur les personnes les moins riches, et les augmenter sur les personnes ou les entreprises les plus riches pour faire jouer la solidarité, et donner à l'Etat les moyens de financer les services publics. (1)* |
| --- | --- |
| **GLOBALIZATION (economic axis)**  Globalization must be framed: international institutions (or even States) must impose rules to better protect the rights of employees, the environment, and the sensitive sectors of each country's economies (such as agriculture or culture). (2)  All customs barriers, along with subsidies or national regulations that distort competition, must be removed so that competition between businesses around the world can take place without hindrance, and in all areas: the most economic efficiency for the benefit of all. (5)  Globalization can be an opportunity: it allows companies to find new markets, and the jobs lost due to offshoring are generally compensated by those which are created, which are more qualified jobs, and which raise the standard of living. But governments must also help their populations when they cannot find their place in globalization. (3)  The globalization of the economy aggravates the exploitation and pollution of poor countries, and causes relocations which destroy jobs in rich countries: truly democratic international institutions must protect the rights of populations (and no longer those of multinationals ) and tax the profits of globalization to help poor countries develop. (1)  Globalization is an opportunity because the opening of borders gives access to new markets, which allows companies to create jobs: it is therefore necessary to break down the "barriers" which prevent products and services from circulating freely; but for national companies to benefit, they must be freed as much as possible from the regulatory constraints that disadvantage them compared to their foreign competitors. (4) | *LA MONDIALISATION (axe économique)* *La mondialisation doit être encadrée : il faut que les institutions internationales (voire les Etats) imposent des règles pour mieux protéger les droits des salariés, l'environnement, et les secteurs sensibles des économies de chaque pays (comme par exemple l'agriculture ou la culture). (2)*  *Il faut supprimer toutes les barrières douanières, en même temps que les subventions ou les réglementations nationales qui faussent la concurrence, pour que la concurrence entre les entreprises du monde entier puisse se faire sans entrave, et dans tous les domaines : c'est de cette façon qu'on obtiendra le plus d'efficacité économique, pour l'intérêt de tous. (5)*  *La mondialisation peut être une chance : elle permet aux entreprises de trouver de nouveaux marchés, et les emplois perdus à cause des délocalisations sont en général compensés par ceux qui sont créés, qui sont des emplois plus qualifiés, et qui font progresser le niveau de vie ; mais il faut aussi que les gouvernements aident leurs populations lorsqu'elles ne trouvent pas leur place dans la mondialisation. (3)*  *La mondialisation de l'économie aggrave l'exploitation et la pollution des pays pauvres, et provoque des délocalisations qui détruisent des emplois dans les pays riches : il faut que des institutions internationales réellement démocratiques protègent les droits des populations (et non plus ceux des multinationales) et il faut taxer les profits de la mondialisation pour aider les pays pauvres à se développer. (1)*  *La mondialisation est une chance, car l'ouverture des frontières donne accès à des marchés nouveaux, ce qui permet aux entreprises de créer des emplois : il faut donc faire tomber les "barrières" qui empêchent les produits et les services de circuler librement ; mais pour que les entreprises nationales en profitent, il faut les libérer le plus possible des contraintes réglementaires qui les désavantagent par rapport à leurs concurrentes étrangères. (4)* |
| POVERTY AND EXCLUSION (identity axis)Rather than assisting people too much (or encouraging them to take advantage of the system), it is necessary to empower them so that they rely more on themselves and less on the State to get by. (5)The state must come to the aid of the most deprived, but we must not expect everything from the state. (3) The state must ensure that everyone receives enough to live decently. (1) | *LA PAUVRETE ET L'EXCLUSION (axe identité)* *Plutôt que de trop assister les gens (ou de les inciter à profiter du système), il faut les responsabiliser afin qu'ils comptent plus sur eux-mêmes et moins sur l'Etat pour s'en sortir. (5)*  *L'Etat doit venir en aide aux plus démunis, mais il ne faut pas tout attendre de l'Etat. (3)*  *L'Etat doit faire en sorte que chacun reçoive de quoi vivre décemment. (1)* |
| PUBLIC SERVICES AND THE PLACE OF THE STATE (economic axis)The State must concentrate its efforts on its main missions of public service and share its other missions with the private sector (for social security, the post office, universities ...) in order to lower its operating costs and gain in efficiency. (4)We must increase the number of public jobs, and devote much more money to public services so that each user, whatever their means, has access to quality public services (for health, education, culture , water, energy, communications, public transport ...); public services have a social mission, they must not seek to be profitable. (1)All public services have a social mission - to leave no one behind - that private companies could not assume; they must have sufficient means to serve the community, but the State must also seek to make them more efficient. (2)To fulfill their mission without representing too heavy a burden on the State, public services must become both more efficient and less costly; some (like, for example, the post or rail transport) can be put in competition with private companies, and even be partly privatized - as long as the State retains control of it - which will encourage them to improve. (3)The State must refocus on its three real missions which are the police, justice and national defense; all the rest can be entrusted to the private sector, whose management methods are much more efficient. (5) | *LES SERVICES PUBLICS ET LA PLACE DE L'ETAT (axe économique)* *L'Etat doit concentrer ses efforts sur ses principales missions de service public, et partager ses autres missions avec le privé (pour la sécurité sociale, la poste, les universités...) afin de faire baisser ses coûts de fonctionnement et de gagner en efficacité. (4)*  *Il faut augmenter le nombre d'emplois publics, et consacrer beaucoup plus d'argent aux services publics afin que chaque usager, quels que soient ses moyens, ait accès à des services publics de qualité (pour la santé, l'éducation, la culture, l'eau, l'énergie, les communications, les transports collectifs...) ; les services publics ont une mission sociale, ils ne doivent pas chercher à être rentables. (1)*  *Tous les services publics ont une mission sociale - ne laisser personne à l'écart - que des entreprises privées ne pourraient pas assumer ; ils doivent disposer des moyens suffisants pour servir la collectivité, mais l'Etat doit aussi chercher à les rendre plus efficaces. (2)*  *Pour assurer leur mission sans représenter une trop lourde charge pour l'Etat, les services publics doivent devenir à la fois plus efficaces et moins coûteux ; quelques-uns (comme, par exemple, la poste ou le transport ferroviaire) peuvent être mis en concurrence avec des entreprises privées, et même être en partie privatisés - dès lors que l'Etat en garde le contrôle - ce qui les incitera à s'améliorer. (3)*  *L'Etat doit se recentrer sur ses trois véritables missions que sont la police, la justice et la défense nationale ; tout le reste peut être confié au privé, dont les méthodes de gestion sont bien plus efficaces. (5)* |
| COMPANIES (economic axis)Social progress must be imposed by law in companies; and the cost of layoffs must be increased for the profit-making companies. (2)The State must give companies back all their freedom, by removing the levies and regulations which are imposed on them and which handicap them in their development. (5)Priority must be given to helping small and medium-sized enterprises by reducing their burdens and administrative constraints, and letting bosses and unions negotiate the operating methods best suited to each branch of activity. (3)Corporate profits must go primarily to employees, and no longer to shareholders; and collective dismissals must be prohibited for companies that make a profit, with the consequence of these companies being requisitioned by the State for the benefit of their employees. (1)Companies need to bear fewer payroll taxes and fewer regulations, so that they are less hesitant to hire and can be more competitive. (4) | *LES ENTREPRISES (axe économique)* *Il faut imposer par la loi des avancées sociales dans les entreprises ; et il faut renchérir le coût des licenciements pour les entreprises qui font des bénéfices. (2)*  *L'Etat doit redonner aux entreprises toute leur liberté, en supprimant les prélèvements et les réglementations qui leur sont imposés et qui les handicapent dans leur développement. (5)*  *Il faut aider en priorité les petites et moyennes entreprises en allégeant leurs charges et leurs contraintes administratives, et laisser patrons et syndicats négocier les modes de fonctionnement les mieux adaptés à chaque branche d'activité. (3)*  *Il faut que les profits des entreprises aillent en priorité aux salariés, et non plus aux actionnaires ; et il faut interdire les licenciements collectifs aux entreprises qui font des bénéfices, sous peine que ces entreprises soient réquisitionnées par l'Etat au profit de leurs salariés. (1)*  *Il faut que les entreprises supportent moins de charges sociales et moins de réglementations, pour qu'elles hésitent moins à embaucher et puissent être plus compétitives. (4)* |
| **THE FIGHT AGAINST DELINQUANCE (identity axis)**  Everyone is responsible for their actions: one can always decide not to fall into delinquency; therefore, to dissuade offenders from committing crime, the sanctions incurred must be really dissuasive. (5)  It is often in difficult contexts that delinquency develops (unemployment, ghettos, family problems, integration difficulties, etc.), but the context does not explain everything; it is a fair balance between prevention and dissuasive sanctions that must be found to fight crime effectively. (3)  Delinquency is first of all the result of difficult contexts (unemployment, ghettos, family problems, integration difficulties, etc.); in order to obtain lasting results in the fight against delinquency, it is therefore in these contexts that priority must be addressed. (1) | ***LA LUTTE CONTRE LA DELINQUANCE (axe identité)***  *Chacun est responsable de ses actes : on peut toujours décider de ne pas tomber dans la délinquance ; aussi, pour dissuader les délinquants de passer à l'acte, il faut que les sanctions encourues soient vraiment dissuasives. (5)*  *C'est souvent dans des contextes difficiles que se développe la délinquance (chômage, ghettos, problèmes familiaux, difficultés d'intégration…), mais le contexte n'explique pas tout ; c'est un juste équilibre entre prévention et sanctions dissuasives qu'il faut trouver pour lutter efficacement contre la délinquance. (3)*  *La délinquance est d'abord le fruit de contextes difficiles (chômage, ghettos, problèmes familiaux, difficultés d'intégration…) ; pour obtenir des résultats durables en matière de lutte contre la délinquance, c'est donc à ces contextes qu'il faut, en priorité, s'attaquer. (1)* |
| **VOTING RIGHTS AND NATIONALITY (identity axis)**  All foreigners, wherever they come from, who have been living in Canada for a long time, must be able to vote at least in local elections; and we must facilitate their acquisition of Canadian nationality. (2)  Only Canadians should have the right to vote; and, with some exceptions, you cannot be Canadian without having Canadian parents: you must apply "blood law", not "soil law". (5)  Only Canadians should have the right to vote; and all people who were born and live in Canada, regardless of their origin, must be Canadian citizens. (3)  All foreigners residing in Canada must have the right to vote, regardless of their nationality. (1)  Only Canadians should have the right to vote; and should only be able to become Canadian if they show their attachment to Canada, by making efforts to integrate, and by making a voluntary process to obtain nationality. (4) | ***DROIT DE VOTE ET NATIONALITE (axe identité)***  *Tous les étrangers, d'où qu'ils viennent, qui sont installés depuis longtemps au Canada, doivent pouvoir voter au moins aux élections locales ; et il faut leur faciliter l'acquisition de la nationalité canadienne. (2)*  *Seuls les Canadiens doivent avoir le droit de vote ; et, sauf exception, on ne peut pas être canadiens sans avoir des parents canadiens: il faut appliquer le "droit du sang", et non le "droit du sol". (5)*  *Seuls les Canadiens doivent avoir le droit de vote ; et tous les gens qui sont nés et qui vivent au Canada, quelle que soit leur origine, doivent avoir la nationalité canadienne. (3)*  *Tous les étrangers résidant au Canada doivent avoir le droit de vote, quelle que soit leur nationalité. (1)*  *Seuls les Canadiens doivent avoir le droit de vote ; et ne doivent pouvoir devenir canadiens que les immigrés qui montrent leur attachement au Canada, en faisant des efforts pour s'intégrer, et en faisant une démarche volontaire pour obtenir la nationalité. (4)* |
| **IMMIGRATION (identity axis)**  Integration works when immigrants feel that they have not only rights, but also duties; and it is important to fight illegal immigration. (4)  The problems linked to immigration do not arise from immigrants, but from the context (economic, social, historical ...) in which immigration occurs, and the first urgency is to ensure respect for the rights of immigrants, whether are in a regular situation or not. (1)  To facilitate the integration of immigrants, it is necessary to fight unemployment, which encourages withdrawal, and to ensure that the rights of immigrants are respected by combating the discrimination to which they may be victims. (2)  For integration to be successful, immigrants must both be less discriminated and respect the values ​​of the host country. (3)  Some immigrants will always remain foreigners: their place would rather be in their country, for our good and for theirs. (5) | ***L'IMMIGRATION (axe identité)***  *L'intégration fonctionne quand les immigrés sentent qu'ils ont non seulement des droits, mais aussi des devoirs ; et il est important de lutter contre l'immigration clandestine. (4)*  *Les problèmes liés à l'immigration ne proviennent pas des immigrés, mais du contexte (économique, social, historique...) dans lequel l'immigration se produit, et la première urgence est de faire respecter les droits des immigrés, qu'ils soient en situation régulière ou non. (1)*  *Pour faciliter l’intégration des immigrés, il faut lutter contre le chômage, qui incite au repli sur soi, et faire respecter les droits des immigrés en luttant contre les discriminations dont ils peuvent être victimes. (2)*  *Pour que l’intégration soit réussie, il faut, à la fois, que les immigrés soient moins discriminés, et qu’ils respectent les valeurs du pays d’accueil. (3)*  *Certains immigrés resteront toujours des étrangers : leur place serait plutôt dans leur pays, pour notre bien et pour le leur. (5)* |
